# Diiron centre mutations in *Ciona intestinalis* alternative oxidase abolish enzymatic activity and prevent rescue of cytochrome oxidase deficiency in flies

**DOI:** 10.1038/srep18295

**Published:** 2015-12-17

**Authors:** Ana Andjelković, Marcos T. Oliveira, Giuseppe Cannino, Cagri Yalgin, Praveen K. Dhandapani, Eric Dufour, Pierre Rustin, Marten Szibor, Howard T. Jacobs

**Affiliations:** 1 BioMediTech and Tampere University Hospital, University of Tampere, FI-33014, Finland; 2 Departamento de Tecnologia, Faculdade de Ciências Agrárias e Veterinárias, Universidade Estadual Paulista “Júlio de Mesquita Filho”, 14884-900 Jaboticabal, SP, Brazil; 3 Institute of Biotechnology, University of Helsinki, FI-00014, Finland; 4 INSERM UMR 1141 and Université Paris 7, Faculté de Médecine Denis Diderot, Hôpital Robert Debré, 48, Boulevard Sérurier, 75019, Paris, France

## Abstract

The mitochondrial alternative oxidase, AOX, carries out the non proton-motive re-oxidation of ubiquinol by oxygen in lower eukaryotes, plants and some animals. Here we created a modified version of AOX from *Ciona instestinalis*, carrying mutations at conserved residues predicted to be required for chelation of the diiron prosthetic group. The modified protein was stably expressed in mammalian cells or flies, but lacked enzymatic activity and was unable to rescue the phenotypes of flies knocked down for a subunit of cytochrome oxidase. The mutated AOX transgene is thus a potentially useful tool in studies of the physiological effects of AOX expression.

The mitochondrial alternative oxidase, AOX, carries out the non proton-motive re-oxidation of ubiquinol by molecular oxygen. Terminal electron transfer by AOX constitutes a parallel system to that provided by OXPHOS complexes III and IV in plants, fungi, protists and many animal phyla^1^. AOX is believed to become activated under stress conditions, when the OXPHOS cytochrome chain is overloaded or unavailable.

In many organisms this is achieved, at least in part, via the regulated expression of the AOX gene, which is induced by a variety of stresses relevant to OXPHOS dysfunction^2^,^3^. The enzyme is also inherently responsive to the metabolic signature of such stresses in different organisms. Firstly, it is activated by high levels of its reduced substrate, ubiquinol^4^,^5^, which is assumed to reflect a lower affinity for the substrate than that exhibited by OXPHOS complex III, with which it competes. Thus, under normal physiological conditions, most of the electron flow from ubiquinol to oxygen is channelled through complexes III and IV, even if AOX is physically present. Only if ubiquinol levels increase, for example, if the enzymatic capacity of complexes III and IV becomes limiting, will AOX become functionally significant. In addition, AOX is allosterically activated in many organisms by metabolites whose levels increase under conditions of OXPHOS insufficiency, for example pyruvate^3^, as well as by other metabolites indicative of cellular redox state.

Although the AOX gene has been lost, during the course of evolution, in the lineages leading to the most complex and advanced metazoan groups, including mammals^1^, we reasoned that its reintroduction by transgenesis should enable such animals to buffer many of the pathological stresses resulting from OXPHOS dysfunction^6^. Thus AOX could become a therapeutic tool for treating mitochondrial diseases and other conditions mediated by OXPHOS dysfunction^7^. Preliminary tests in model organisms, including cultured human cells^8^,^9^, *Drosophila*^10^,^11^ and the mouse^12^, support this concept. In particular, the expression of AOX from the tunicate *Ciona intestinalis*, was shown to compensate many of the phenotypes resulting from cytochrome oxidase (COX, complex IV) deficiency in *Drosophila*, including the knockdown of structurally essential subunits of the complex^11^. However, if AOX is to be of value in eventual therapy, the mechanism of this compensation needs to be established. The hypothesized enzymatic by-pass is only one of several possible such mechanisms. Expression of an inert transgene, such as GFP, in place of AOX, was unable to rescue the phenotypes produced by engineered deficiency of cytochrome oxidase^10^,^11^. However, this control cannot be unambiguously interpreted, since the expressed GFP was not targeted to mitochondria, and even if it were, does not possess other structural features of AOX that enable it to insert into the inner mitochondrial membrane in a specific fashion and interact with other components thereof.

In order to provide a more applicable test of whether the ability of AOX to rescue COX deficiency depends on its primary enzymatic activity, we sought to engineer the AOX in such a way as to destroy this activity, whilst producing only a minimal effect on the overall structure, stability and expression of the protein. To do this, we took advantage of the fact that AOX is well conserved phylogenetically, that the residues contributing to its active site have been characterized in a number of species, and that the structure of a representative AOX, from the protistan parasite *Trypanosoma brucei*, has recently been published^13^. Using currently available bioinformatics tools, we modelled the structure of the *Ciona intestinalis* enzyme against this template, predicted amino-acids required for binding the catalytically essential diiron moiety at the active site, and proceeded via alanine-substitution mutagenesis to create an expressible version of the enzyme expected to lack enzymatic activity, despite being predicted to fold to a similar overall structure. In several different contexts (cultured human and *Drosophila* cells, as well as whole animals), we found that the mutated AOX was stably expressed but devoid of detectable enzymatic activity. Furthermore, expression of the transgene encoding the mutated AOX was unable to rescue engineered COX deficiency in the fly, confirming that this rescue indeed depends on the enzymatic activity of AOX.

## Materials and Methods

### Sequence alignments and molecular modelling

The sequences of AOX homologues found by BlastP searching were aligned using the MUSCLE algorithm built into the software MEGA6^14^, with default parameters. A homologous model of the structure of one subunit of the *C. intestinalis* AOX was generated using the software I-TASSER^15^, based on the crystal structure of the *Trypanosoma brucei* AOX (PDB 3VV9:A)^13^ as template and the multiple sequence alignment described above as input restraint. Other parameters were set as default. Selection of the model was based upon the best accuracy estimations provided by the C-scores, estimated TM-scores and RMSD values. Because the N-terminal region (M1-K103) of the *C. intestinalis* AOX structure could not be modelled with high accuracy, this region was eliminated from the analysis. The dimeric model of *C. intestinalis* AOX and the positioning of the two diiron centres (one per subunit) were built by overlapping two copies of the model generated by I-TASSER into the crystal structure of the dimeric *T. brucei* AOX using Pymol (www.pymol.org). Pymol was also used to analyze all structure models and to produce the figures.

### Cloning procedures and mutagenesis

For *Drosophila* expression, the *C. intestinalis* AOX coding sequence, including its natural stop codon, was recloned from the pMT/V5-His B vector (Invitrogen), in which it had been previously propagated, into the *Eco*RI site of pUASTattB^16^. Based on the multiple sequence alignment shown in Fig. S1, and the results of molecular modelling (see Results), PCR-based alanine substitution mutagenesis and recloning were carried out according to the scheme of Fig. S2. Mutations E239A, H242A, E344A and H347A were introduced, using the plasmid-borne AOX cDNA as template, Pfu DNA polymerase (Stratagene) and oligonucleotides (all shown 5′ to 3′) as follows: GAAGCTGAAAATGcGAGAATGgcCTTAATGACTGCG and CGCAGTCATTAAGgcCATTCTCgCATTTTCAGCTTC to create E239A/H242A, followed by ATCTGAGCTGATGcAGCACATgcCAGATCAGTCAAC and GTTGACTGATCTGgcATGTGCTgCATCAGCTCGGAT to create E344A/H347A (lowercase letters indicate the sites of introduced mutations). For expression in S2 cells, constructs containing the original and mutated AOX cDNA inserts, again using the natural stop codon, were recloned into the *Eco*RI site of pAc5.1/V5-His B (Invitrogen, USA) to create pAC/AOX^17^ and pAC/mutAOX. For transient mammalian expression, the wild-type and mutated AOX coding sequences were recloned, respectively, into a pBR322-derived *kan*^*R*^ plasmid containing the CAG promoter^18^ and bovine growth hormone poly(A) signal, together with other elements not relevant to the present study (copies of the *tet* operator, *loxP* sites, insulator elements and portions of the porcine *Ggta1* gene), to create the expression constructs pCAG-AOX and pCAG-mutAOX. The nucleotide sequences of all clones were confirmed by Sanger sequencing using the Big Dye Terminator v3.1 kit (Life Technologies) and an ABI3130xl Genetic Analyzer, according to the manufacturer’s specifications.

### Drosophila stocks and maintenance

Except where stated, flies were maintained and grown on standard medium at 25°C, using a 12 h light/dark cycle, as previously^10^,^19^. Balancers, recipient line *w*^*1118*^, the RNAi line for CG9603 (Vienna Drosophila RNAi Center line 106661), the ubiquitous *da-GAL4* driver (Bloomington line 8641) and the driver line bearing *elav*^*C155*^*-GAL4* on chromosome X and UAS*-Dcr2* on chromosome 2 (Bloomington line 25750), were obtained from stock centres. ΦC31 recombinase-mediated-site-directed transgenesis was used to generate transgenic fly lines (service provided by BestGene Inc, Chino Hills, CA), using recipient lines with the following integration sites: *attP18* (chromosome X), *attP40* (chromosome 2) and *attP2* (chromosome 3), according to Pfeiffer *et al.*^20^, employing the wild-type and mutated AOX constructs cloned in pUASTattB and pUASTattB itself as empty-vector control. Following characterization, transgenic lines were maintained over balancers appropriate for chromosome X, 2 or 3, bearing standard markers (FM7, CyO, TM3Sb, respectively). Transgenic lines UAS-AOX^F24^ and UAS-AOX^F6^ were described previously^10^.

### Cell culture and transfection

HEK293T cells were cultured as previously^21^. Plates of 3 × 10^6^ cells were transfected with 24 μg of the pCAG-AOX or pCAG-mutAOX plasmids or, as control, empty vector (pWPI, Addgene), using 60 μl Lipofectamine® 2000 (Invitrogen) under manufacturer’s recommended conditions*. Drosophila* S2 cells were grown and transfected with pAc5.1/V5-His B or derivatives as previously^17^.

### Expression assays

RNA extraction and QRTPCR to measure AOX transcript levels using *RpL32* RNA as an internal normalization standard were as previously described^10^, using RNA from 2 day-old adult male and female flies. Protein extraction from 2 day-old *Drosophila* adults and Western blots were conducted essentially as by Fernandez-Ayala *et al.*^10^, with the following modifications: for females, 1% SDS was used for lysis instead of 1.5% Triton X-100, flies were processed in batches of 30 (females) or 40 (males), SDS-PAGE used Any kD™ Criterion™ TGX™ 18-well gels (Bio-Rad), Prestained Protein Ladder (Thermo-Scientific) and ProSieve^TM^ EX Running and Transfer Buffers (Lonza), and membranes were treated in PBS-Tween® instead of TBS. Primary antibodies used were customized rabbit anti-AOX^10^ (21^st^ Centrury Biochemicals, 1:10,000), rabbit anti-α-actininin C-20-R (Santa Cruz Biotechnology, 1:5,000) and mouse anti-ATP5A (Abcam, 1:50,000). Secondary antibodies were Peroxidase Goat Anti-rabbit IgG and Horse Anti-mouse IgG (both from Vector Laboratories, 1:10,000). Post-nuclear extracts (PN) from HEK293T cells were prepared according to Cannino *et al.*^21^. Protein concentrations were measured using the Bradford assay.

### Respirometry

Oxygen consumption of 5 × 10^6^ human cells was measured 48 h after transfection, following permeabilization with 80 μg/ml digitonin, in a Clark-type electrode (Hansatech Oxytherm system) using respiratory buffer A^22^ at 37 °C. Complex II-driven respiration was measured in the presence of 10 mM ADP and 10 mM succinate. AOX-driven (antimycin-resistant) respiration was measured after the further addition of (60 ng/ml) antimycin A, with subtraction of any residual oxygen consumption after adding 100 μM *n*-propyl gallate. Respirometry on S2 cells was as described previously^17^ and was also conducted on homogenates from 1–4 day-old *Drosophila* males. Briefly, 25 males were gently homogenized in 0.8 ml ice-cold isolation buffer (250 mM sucrose, 5 mM Tris-HCl, 2 mM EGTA, pH 7.4) and muslin-filtered. Respirometry was performed on 150 μl aliquots of this homogenate, mixed with 500 μl assay buffer (120 mM KCl, 5 mM KH_2_PO_4_, 3 mM HEPES-KOH, 1 mM EGTA, 1 mM MgCl_2_, 0.2% BSA, pH 7.2), substrates (15 mM glycerol-3-phosphate and 5 mM ADP) and inhibitors as for permeabilized mammalian cells.

### Behavioural assays

Time to eclosion following *Drosophila* crosses was measured as previously^23^. Eggs from parents crossed two days earlier were collected over three consecutive nights, and cultured at 25°C. Adults less than 24 h old were collected and sorted on ice, after which batches of 5 male flies were placed in each empty vial. After a 10 min waiting period, flies were tipped down and their subsequent behaviour recorded using a DFK 21AF04 camera (The Imaging Source, Bremen, Germany) and Media Recorder 2 software (Noldus, Wageningen, Netherlands). The climbing index^11^ for each vial was manually calculated from recordings as the mean number of flies which climbed 6 cm in 10 s in three trials. Climbing indices from different genotypes were compared by one-way ANOVA with Bonferoni adjustment, using SPSS 12. The box plot was drawn with BoxPlotR (boxplot.tyerslab.com), with Tukey style whiskers extending to the data point that is no more than 1.5 × IQR (interquartile range) from the edge of the box^24^.

### Human subjects

The work reported here did not use human subjects or any materials derived from human subjects, other than the freely available cell-line HEK293T.

## Results and Discussion

### Modelling and creation of mutated AOX transgene

**Figure 1:**
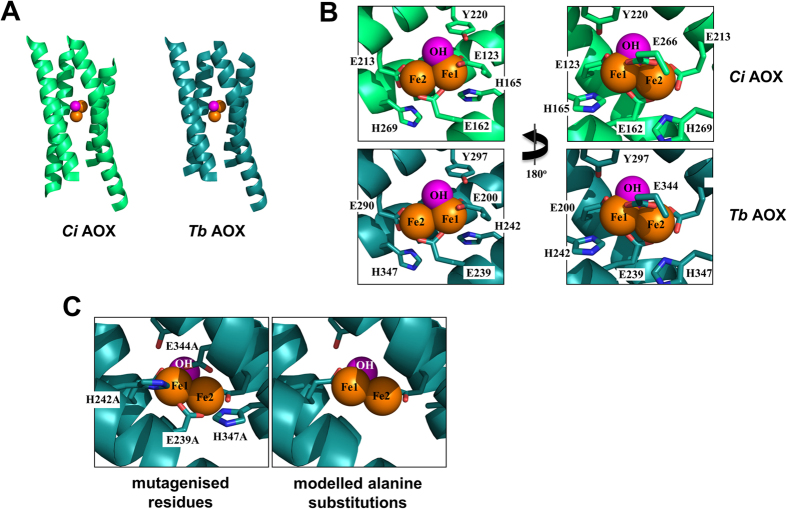
Structural modelling and mutagenesis of active site of *Ciona intestinalis* AOX. (**A**) Model of the active site of the *Ciona* (*Ci*) enzyme, green, compared with the structure of the *Trypanosoma brucei* (*Tb*) AOX, blue. In both cases, the diiron site (iron moieties in orange, hydroxyl in pink) is buried in a four alpha-helix bundle. For clarity, only one protomer is shown. (**B**) Conserved residues binding the diiron centre show an identical arrangement in the *Ci* model (green) as in the *Tb* structure (blue). (**C**) The residues selected for alanine-substitution mutagenesis in the *Ci* enzyme (here shown in blue), alongside the resulting modelled structure.

Alignment of the predicted *Ciona intestinalis* AOX amino-acid sequence with the corresponding protein from other taxa, including *Trypanosoma brucei*, revealed conservation of residues implicated in the organization of the diiron centre of the enzyme, as previously reported by Shiba *et al.*^13^. The four invariant glutamate residues and two histidines correspond in *Ciona* AOX with E200, E239, E290, E344, H242 and H347 (Fig. S1), numbered from the first methionine of the putative preprotein. In the *Trypanosoma* AOX structure, the conserved histidines participate in a hydrogen bond network that also includes a conserved tyrosine, Y297 in *Ciona* AOX (Fig. S1). Structural modelling (Fig. 1) showed that *Ciona* AOX can fold to an almost identical structure as its *Trypanosoma* counterpart, ignoring the poorly conserved N-terminal region (residues 1–103 of the *Ciona* protein, Fig. S1). Four alpha-helices enclose the diiron centre of each protomer of the homodimeric protein, with the conserved glutamate and histidine residues similarly juxtaposed as in the *Trypanosoma* protein (Fig. 1). Based on this structure, we tested the functional significance of the conserved residues at the predicted diiron centre, by mutating four of them to alanine (E239A, H242A, E344A, H347A), in appropriate transgenic constructs for expression in mammalian cells and *Drosophila* (Fig. S2). The mutations were predicted to destroy the binding of iron to the active site, whilst only minimally disturbing the overall structure of each subunit.

### Mutated AOX can be stably expressed in mammalian cells and flies

**Figure 2:**
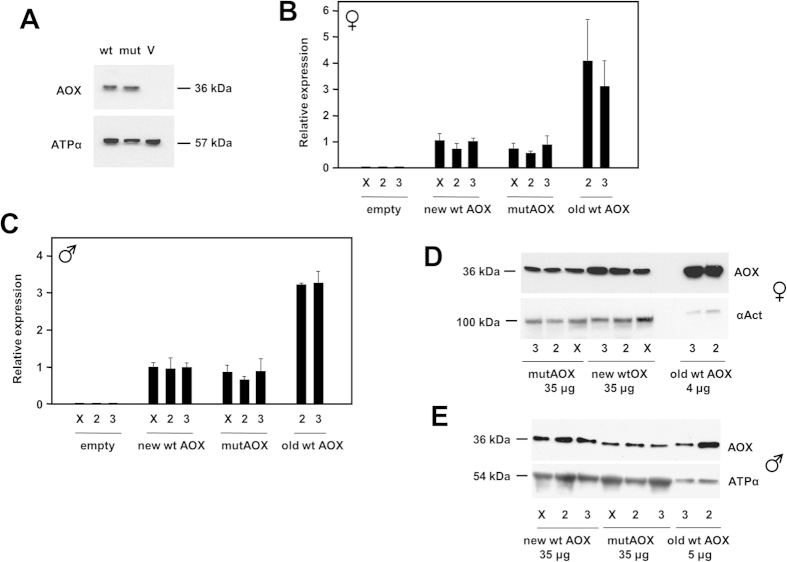
Expression of AOX transgenes in mammalian cells and *Drosophila*. (**A**) Western blot of protein extracts from HEK293T cells transfected with wild-type and mutated AOX constructs (wt, mut) or empty vector (V), probed for AOX and for ATP synthase subunit α as loading control. (**B,C**) Relative AOX expression at RNA level, based on QRTPCR, in (**B**) females and (**C**) males of different *Drosophila* lines transgenic for wild-type or mutated AOX, or empty vector, inserted on chromosomes X, 2 and 3, as shown, in combination with the ubiquitous *da*-*GAL4* driver. New wt (wild-type) and mutAOX lines were those created by site-specific integration at defined chromosomal sites using the ΦC31 system; old wt AOX lines were UAS-AOX^F6^ (chromosome 2) and UAS-AOX^F24^ (chromosome 3). For males, all values were significantly different from empty-vector lines; old wt AOX lines were significantly different from new wt AOX lines (*p* < 0.001, ANOVA followed by post-hoc Bonferoni-corrected *t* test), but mutAOX and new wt AOX lines were not significantly different from each other. Statistical analysis for females gave similar results, although greater sample-to-sample variation for old wt AOX lines yielded only *p* < 0.05 comparing them with new wt or mutAOX lines. (**D,E**) Western blot of protein extracts from the same flies (amounts as shown), probed for AOX or, as loading control, either ATP synthase subunit α or α-actinin, as indicated.

In order to test its functionality, the expression of the mutated AOX construct (mutAOX) was first verified, following transient transfection into cultured human cells. Based on Western blotting (Fig. 2A), the mutAOX protein was the same size and comparably expressed as wild-type AOX. Next, the mutAOX transgene, under the control of the GAL4-dependent UAS promoter, was introduced into the *Drosophila* genome by targeted insertion at single sites on each chromosome. Parallel control lines were created, containing wild-type AOX and empty vector, inserted at the same sites. Following validation of the insertions by PCR and sequencing, we measured transgene expression directed by the ubiquitous *da*-*GAL4* driver, at both RNA and protein levels, using QRTPCR (Fig. 2B, C) and Western blotting (Fig. 2D, E).

In both females (Fig. 2B) and males (Fig. 2C), the expression of wild-type and mutAOX were similar at the RNA level, but 3–4 fold less than AOX in the previously created transgenic lines, engineered by random P-element insertion. At the protein level, mutAOX showed slightly lower expression than wild-type AOX in both sexes, and expression was again less than in the previously created lines (Fig. 2D, E).

**Figure 3:**
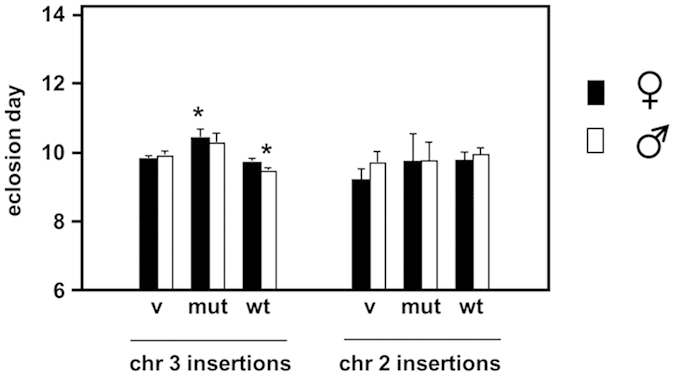
Developmental time to eclosion of AOX transgenic flies. Eclosion day (mean +SD) for females and males of different *Drosophila* lines transgenic for wild-type (wt) or mutated (mut) AOX, or empty vector (v), inserted on chromosomes X, 2 and 3, as shown, in combination with the ubiquitous *da*-*GAL4* driver. *denotes significant difference from flies of the same sex from the empty vector line on the same chromosome, *p* < 0.05 (Student’s *t* test).

When expressed ubiquitously using the *da*-*GAL4* driver, the AOX and mutAOX transgenes produced only very small changes in developmental timing, most of them non-significant compared with the corresponding vector-only line (Fig. 3).

### Mutated AOX lacks detectable enzymatic activity

**Figure 4:**
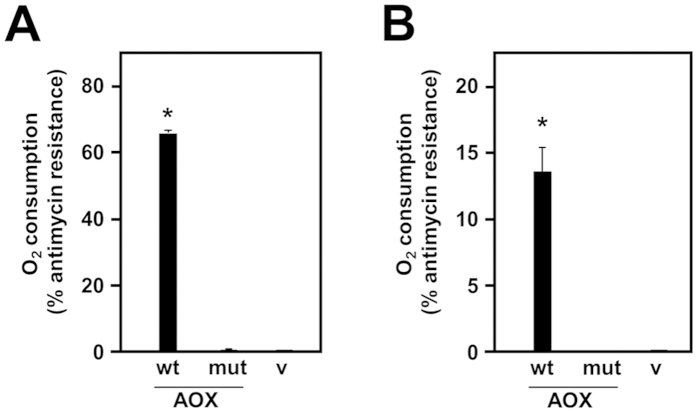
Respirometry of AOX-transfected cells and flies. Oxygen consumption (% resistant to antimycin, as defined in Materials and Methods) of (**A**) permeabilized, transiently transfected cells, and (**B**) homogenates from male transgenic flies induced for expression using *da*-*GAL4* driver, expressing wild-type (wt) or mutated (mut) AOX or empty vector (v). The flies had transgenic insertions on chromosome 2. *denotes significant difference from vector-only flies.

The functionality of the expressed AOX variants was tested by polarography. Permeabilized HEK293T cells, following transient transfection with wild-type AOX, supported approximately 80% of the uninhibited oxygen consumption, in the presence of antimycin. Antimycin-resistant oxygen consumption was undetectable in permeabilized cells transiently transfected with the mutAOX construct or empty vector (Fig. 4A). A similar result was obtained after transfection of *Drosophila* S2 cells. After transfection with either of two different AOX-expressing constructs, whole-cell respiration in the presence of antimycin was 70–73% of the uninhibited rate, but was undetectable in control cells or cells transfected with the mutAOX construct (Table S1). Finally, in homogenates from male transgenic flies carrying targeted insertions at the same locus (on chromosome 2), induced to express the transgene ubiquitously using the *da*-*GAL4* driver, wild-type AOX supported 14% of the uninhibited substrate oxidation rate in the presence of antimycin (Fig. 4B), whereas mitochondria from mutAOX- or empty vector-transgenic flies showed no antimycin-resistant substrate oxidation. In every polarography experiment, expression of the AOX transgene was verified by Western blotting as per Fig. 2.

### Mutated AOX is unable to rescue COX knockdown in flies

The fact that the mutated AOX is devoid of detectable enzymatic activity allowed us to use the newly created transgenic lines to test whether the previously observed phenotypic rescue of flies knocked down for a subunit of cytochrome oxidase (Cox7a) was due to the enzymatic activity of AOX or some other property conferred by the AOX protein, when expressed in *Drosophila*. Moreover, the fact that the newly created transgenic lines express AOX at only about 30% of the level of the lines previously studied, allowed us to test whether phenotypic rescue was quantitatively dependent on AOX expression level. Ubiquitous knockdown of CG9603, the broadly expressed isogene for Cox7a, was previously shown to produce pupal lethality^11^, which was rescued by high-level expression of AOX.

**Figure 5:**
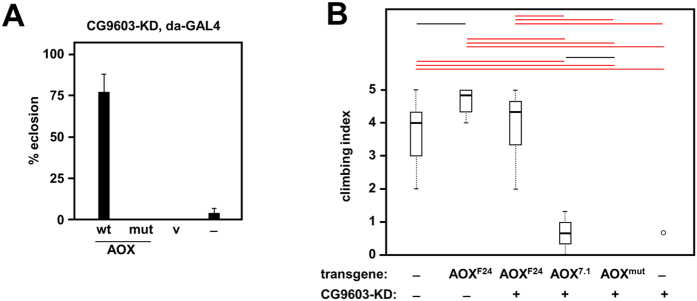
AOX rescue of Cox7a deficiency. (**A**) Survival (%) from egg to eclosion of flies of the indicated genotypes, all bearing the *da*-*GAL4* driver and the CG-9603 knockdown (RNAi) construct. Lines tested contained either no additional transgene (–), vector only (v), wild-type (wt) or mutated AOX (mut), in each case on chromosome 3. (**B**) Boxplot of climbing index of flies of the indicated genotypes. All flies carried the *elav*^*C155*^-*GAL4* driver on chromosome X plus UAS-*Dcr2* with or without the CG9603 knockdown (RNAi) construct on chromosome 2, and the indicated AOX transgene on chromosome 3 (AOX^7.1^ is the ΦC31-targeted insertion). Bars indicate medians, boxes show the first and third quartiles percentiles, whiskers are plotted according to the Tukey scheme (Krzywinski and Altman, 2014). Significant differences based on ANOVA are indicated by horizontal lines (black, red) denoting *p* < 0.05 and 0.001, respectively. A single outlier point is indicated by an open circle.

To test the new transgenic lines, we first confirmed that the RNAi line used in the experiment was devoid of the additional insertion previously reported to confer pupal lethality unrelated to specific target knockdown^25^ (Fig. S3). We then combined the CG9603 RNAi line with AOX and control transgenes, plus the *da*-*GAL4* driver to induce simultaneous transgene expression and Cox7a knockdown. Wild-type AOX rescued the lethality, as previously (Fig. 5A), whereas mutAOX or the empty vector were unable to do so, confirming that AOX enzymatic activity is required for the rescue.

Next, we investigated the effects of CG9603 knockdown and its potential rescue by AOX, using the neuron-specific driver *elav*^*C155*^-*GAL4*. Previously, it was shown that this produces a locomotor defect in newly eclosed flies^11^. To potentiate the phenotype, we included *UAS*-*Dcr2* in the background, so as to increase the penetrance of RNAi. Without concomitant AOX rescue, the resulting flies showed a severe locomotor defect as measured by their inability to climb the walls of the vial, in a standard negative geotaxis assay (Fig. 5B). High-level expression of AOX produced, as before, a clear rescue, whilst lower-level expression using the newly created transgenic lines produced only a modest phenotypic improvement (wild-type AOX), or no improvement at all (mutAOX, Fig. 5B).

### Structural conclusions

Alternative oxidases are members of a superfamily of metalloenzymes, characterized by a common catalytic function of activation of molecular oxygen, and by common structural elements defining the catalytic diiron centre, including the four-helix bundle fold and a motif comprising two histidine residues, four carboxylate groups, and a bridging carboxylate group across the diiron centre^26^,^27^,^28^. The crystal structure of the trypanosomal enzyme indicates that it is a homodimer with each monomer comprising six long and four short α-helices^13^. The subunits interact with each other via α-helices 2, 3 and 4, whereas the hydrophobic region formed by α-helices 1, 2, 4 and 5 is proposed to anchor the protein to the inner surface of the mitochondrial inner membrane. A series of conserved arginine residues, capable of interacting with phospholipid head-groups, may assist inner membrane anchorage^13^. Our structure modelling of the *C. intestinalis* AOX suggests that the same structural elements are conserved in animal AOXs, and that the enzyme is also a homodimer inserted into the mitochondrial inner membrane.

In addition, the model predicts that the active site, and therefore the mechanism of oxygen activation, are also conserved in animal AOXs. The four-helix bundle, which acts as a structural platform for the binding of the two iron atoms, buries the active site deep in a hydrophobic environment. In *T. brucei* AOX, glutamate residues 123, 162, 213 and 266, in addition to a hydroxo-bridge, are responsible for directly coordinating the diiron centre. The centre is further stabilized by a redox-active tyrosine residue^29^,^30^, Y220, and two histidine residues (H165 and H269), which are within hydrogen-bond distances of E123, E169 and E213. The *C. intestinalis* AOX model indicates that the homologous residues E200, E239, E290, E344, Y297, H242 and H347 organize the active site in the same way.

### Functional conclusions

In theory, the mutagenesis of a single glutamate residue should be enough to destabilize the diiron centre^31^. However, taking advantage of the proximity in the DNA sequence of the codons for E239 and H242 and of those for E344 and H347, we were able to create alanine substitutions for four important active site residues simultaneously. According to our model, these mutations should disrupt iron binding, thus generating a mutant devoid of catalytic activity, without any major disturbance to the overall protein structure. These predictions are supported by the fact that the mutant and wild-type proteins were expressed at comparable levels in mammalian cells and in flies, but that no enzymatic activity could be detected.

Importantly, the mutated enzyme was unable to rescue the organismal phenotypes arising from engineered cytochrome oxidase deficiency. In theory, the action of a foreign protein in attenuating such phenotypes could be due to any of several different mechanisms, of which the provision of an enzymatic by-pass for ubiquinol oxidation is only one. In previous work we found that *Ciona* AOX, when expressed in *Drosophila* mitochondria, decreased the net production of mitochondrial ROS even under non-inhibited conditions^10^,^32^. The mechanism of this remains unknown, but one possibility is that AOX is able to act directly or indirectly as an antioxidant, e.g. by binding and quenching quinone radicals via some other mechanism. Studies in various organisms have supported the idea that a hydrophobic pocket, located between α-helices 2 and 3, binds and channels ubiquinone to the active site^33^, which might be involved in such an activity.

A second possibility would be a hormetic response to disruption of the inner mitochondrial membrane or its protein complexes by the foreign protein. The induction of a variety of defence pathways to protect cells from increased ROS, disturbed protein, lipid or redox homeostasis, or altered mitochondrial turnover or dynamics, might equip the organism to cope with the additional but related stresses of respiratory insufficiency. Many studies in model organisms support this concept of ‘mitohormesis’^34^. Whilst we cannot rule out that such effects are material in other contexts, our findings do exclude them in regard to the developmental lethality produced by global cytochrome oxidase knockdown, or the locomotor dysfunction resulting from its knockdown specifically in neurons^11^. Based on our findings, that mutAOX cannot compensate these phenotypes, we infer that the rescue of these effects of cytochrome oxidase deficiency by AOX is almost certainly due to its enzymatic activity as a quinol oxidase, though formally we cannot exclude other, unknown effects of iron binding. A requirement for enzymatic activity might not be true of every phenotypic feature conferred by AOX in model organisms. Our findings indicate a robust way to test this in regard to all potential such phenotypes, allowing the mechanisms by which AOX acts to be probed, controlled or verified.

Several quantitative issues are also addressed by our findings. The first is that the extent of phenotypic rescue depends in some instances on the AOX expression level, but in other cases, such as the rescue of the developmental lethality caused by ubiquitous COX knockdown, is an all-or-none phenomenon. We suggest that this reflects a threshold effect wherein even the three-fold lower expression level of AOX, when integrated at specific sites by ΦC31-mediated recombination (in comparison with P element-mediated integrants created previously), exceeds a threshold value required to maintain metabolic homeostasis and complete development. In contrast, the lower expression level of the targeted integrants gave a clearly weaker rescue of locomotor dysfunction, when COX was knocked down only in neurons, roughly in proportion to the decreased expression level.

It may also be noted that the amount of antimycin-resistance conferred upon respiration in homogenates from the targeted integrants was still approximately 14%, compared with approximately 20% for the P element-mediated integrants, even though they are expressed at a much higher level. The level of respiratory antimycin-resistance in the fly may vary between tissues, and this 20% maximum may reflect only the properties of the predominant class of mitochondria. Most of the respiratory capacity in adult flies is vested in the flight muscles, where mitochondria make up almost one-third of the total tissue mass^35^. The apparent upper limit of how much electron flow can be diverted through AOX probably reflects specific features of this tissue and its energetic needs. The limit could be dictated by the constraints of membrane architecture, for example, if much of the ubiquinone pool is channelled directly from complex I to complex III via respiratory supercomplexes, such that it equilibrates only slowly with free ubiquinones available to AOX^36^. Most of the respiratory activity in adult *Drosophila* indeed resides in supercomplexes^37^. Such a phenomenon may account for the inferred threshold effect on the rescue of developmental lethality. Conversely, the organization of the respiratory chain may differ in other tissues, such as in neurons, where a more graded response to the AOX expression level is evident.

In conclusion, mutAOX offers a useful tool for future studies of the mechanism(s) whereby expression of *Ciona* AOX modifies the phenotypes of model organisms, potentially contributing the eventual development of AOX-based therapies.

## Additional Information

**How to cite this article**: Andjelković, A. *et al.* Diiron centre mutations in *Ciona intestinalis* alternative oxidase abolish enzymatic activity and prevent rescue of cytochrome oxidase deficiency in flies. *Sci. Rep.* **5**, 18295; doi: 10.1038/srep18295 (2015).

## Supplementary Material

Supplementary Information
