# Supplementary material for: Diiron centre mutations in Ciona intestinalis alternative oxidase abolish enzymatic activity and prevent rescue of cytochrome oxidase deficiency in flies
Source: Sci Rep. 2015 Dec 17;5:18295. doi: 10.1038/srep18295 (PMC4682143; doi:10.1038/srep18295)
Supplement: Supplementary Information [file srep18295-s1.pdf]

Diiron centre mutations in *Ciona intestinalis* alternative oxidase abolish enzymatic activity and prevent rescue of cytochrome oxidase deficiency in flies

Ana Andjelković, Marcos T. Oliveira, Giuseppe Cannino, Cagri Yalgin,

Praveen K. Dhandapani, Eric Dufour, Pierre Rustin, Marten Szibor and Howard T. Jacobs

SUPPLEMENTARY DATA

SUPPLEMENTARY TABLE

Table S1

Oxygen consumption rate of transfected S2 cells[1]

Treatment	wt AOX (pAC)	wt AOX (pUAST)	mutAOX	untransfected
cells only	22.5	17.4	22.6	19.4
+ antimycin	16.3	13.1	1.8	1.9
+ nPG	1.6	1.5	1.8	2.5
% antimycin-resistant[2]	70	73	0	0

[1]pmol.s^{-1}.ml^{-1} from a representative experiment

[2]calculated as 100 times [the oxygen consumption rate in the presence of antimycin – the oxygen consumption rate in the presence of antimycin plus n-PG], divided by [the uninhibited oxygen consumption rate antimycin – the oxygen consumption rate in the presence of antimycin plus n-PG].

LEGENDS TO SUPPLEMENTARY FIGURES

Figure S1

Amino acid sequence alignment of eukaryotic mitochondrial alternative oxidase. The selected sequences were retrieved from NCBI Protein database using the sequence of *Ciona intestinalis* AOX as query in BlastP searches. The alignment was performed using the MUSCLE algorithm built in the software MEGA6 with default parameters. Residues highlighted in green form α-helices in the crystal structure of *Trypanosoma brucei* AOX; the ones highlighted in red are responsible for coordinating the binding to the diiron cluster. The last data line indicates invariant amino acids in the taxa shown (*). Note that some of the sequences compiled from genome assemblies are probably incomplete (e.g. *Trichoplax adhaerens*, *Nematostella vectensis*).

Figure S2

Cloning and mutagenesis strategy. The AOX coding region (red), including its natural stop codon, was amplified from the original vector pMT/V5-His B (abbreviated as pMTb) and recloned into the *Eco*RI site of pUASTattB for site-specific integration into the *Drosophila* genome. Separately, the original plasmid was used for PCR-based *in vitro* mutagenesis (see main text) as indicated (substitutions in green), to create the mutated AOX (mutAOX) coding sequence (green). This was then recloned into ZeroBluntTOPO for use in other systems, and thence into pUASTattB to create the construct for transgenic integration in parallel to the wild-type cDNA.

Figure S3

Verification of transgenic insertion status in VDRC RNAi lines. Green et al (2014) reported

that some lines in the VDRC RNAi collection contain additional insertions, conferring pupal lethality and other phenotypes when activated, due to insertional effects. Insertions at the desired (‘good’, NA, non-annotated) site can be distinguished from those at the undesired (‘bad’, A, annotated) site by simple PCR-based analysis specific for the two sites, as recommended by Green et al (2014). In each case, the insertion is distinguished by gel-mobility difference, indicating whether the site is occupied or unoccupied. In the trials shown here, we tested VDRC line 106661 (targeted on CG9603), using as controls the parental line 60100 with insertions at neither site, and line 109338 (targeted on CoIV), which carries insertions at both sites. Line 106661, to be used in the experiments described here, is thus verified to have only the desired insertion, as indicated.

```
Ciona intestinalis                  MLSTGSKTFLFRPFLGSCHALQSGKLPCSNLHTTP------------TKI 38
Strongylocentrotus purpuratus       -----------------------MEVRSKDTLAPP--------------- 12
Crassostrea gigas                   --MGSLRQITKLSENGVRIFCSQLKNLENNSILLR--------------- 33
Urechis unicinctus                  -MMARVTVRLLLAKDSHTILSQAVRQMIPYAASHN--------------- 34
Nematostella vectensis              --------------------------------------------------
Trichoplax adhaerens                --------------------------------------------------
Amphimedon queenslandica            --MATSVWLRSNSRQGNFIY---TRFISAGKCHRS--------------- 30
Penicillium rubens                  --MNTLSVRAPLRAAARPQY---LHLAVRTYSGVV--------------- 30
Aspergillus flavus                  ---------------MASFF---LNITCPNRACLA--------------- 17
Arabidopsis thaliana                MMITRVEPRAQIAVSGGWTT---FVLDGPYVSSHE--------------- 32
Nicotiana tabacum                   -----------------MWV---RHFPVMGP------------------- 11
Zea mays                            -MSTRAAGSALLRHLGPRVF---GPVFSPAVAPPR--------------- 31
Acanthamoeba castellanii            MKQHCSQRIASLRGGGRDAF---ARLATTTASSLASGNGGVRASTLAQAR 47
Gregarina niphandrodes              ---MALINQLVSRAALRPLN---VRAISKSPLRPD--------------- 29
Tetrahymena thermophila             MRANLFKKCLQIHKNTNTLFSVSRRFKSDLQYTPE--------------- 35
Paramecium tetraurelia              --------------------------------------------------
Trypanosoma brucei                  ---MFRNHASRITAAAAPWV---LRTACRQKSDAK--------------- 29


Ciona intestinalis                  TVKRYLVGYSWSTQPHSRLLHSCQQLKIDDKNKSEHFKIETNDSTDEPNI 88
Strongylocentrotus purpuratus       ----------------------------LKHKHQEKLMVKKSQLLLHTSK 34
Crassostrea gigas                   ---------------VSGIRTSNGLRNAGTKADVDENIKKFKEENFEKIP 68
Urechis unicinctus                  -ALITSMPQVYAYSTQTRSLNNKAKESVNLGPHIQENLKKFREGSHENVS 83
Nematostella vectensis              --------------------------------------------------
Trichoplax adhaerens                --------------------------------------------------
Amphimedon queenslandica            ----------------------------------------VRAFSASSNE 40
Penicillium rubens                  ---------------------------ATTLNSSCVVSKRTSAFSLTSKR 53
Aspergillus flavus                  --------------------------------------AGNSAQLLGKHV 29
Arabidopsis thaliana                -----ALSRSHILKPGVTSAWIWTRAPTIGGMRFASTITLGEKTPMKEED 77
Nicotiana tabacum                   ----------------------------------------RSASTVALND 21
Zea mays                            ----------PLLALAGGGERGGALVWVRVRLLSTSAAEAKEEVAASKGN 71
Acanthamoeba castellanii            RHLSLRLAPTTMTSTTSRTSSATSTTMTTRGRWCQGGALAWSRANTTSAA 97
Gregarina niphandrodes              -----------------RILWTDFRMPSTAAQTRRLPQFTEQRRTVVFKK 62
Tetrahymena thermophila             ----------------------------------------NNFFQNTFNS 45
Paramecium tetraurelia              -----------------------------------------------MNR 3
Trypanosoma brucei                  -------------------------------------TPVWGHTQLNRLS 42


Ciona intestinalis                  EVENFPHFREAKKAKETQKGSSLAEAEEHPDVEEGRAMQDGGYRLPHPIW 138
Strongylocentrotus purpuratus       ESGKVDDHIQAAVEKPGSQ----------------------KYLLPHPIW 62
Crassostrea gigas                   DPEQLDHFRKTQSTDQLVESMKN-------------PPPMGTHTLPHPIW 105
Urechis unicinctus                  VPEELQHFRKSTEEGVKGNGPEEE------------KPPMGAVALPHPIW 121
Nematostella vectensis              --------------------------------------------------
Trichoplax adhaerens                --------------------------------------------------
Amphimedon queenslandica            PEEKAPHFKRSSVVHPLSAHIKM-------------VMQEKPYTLPHPIW 77
Penicillium rubens                  PISSTPKSQTITDYFPAPETP---------------NVKEVQTAWVHPVY 88
Aspergillus flavus                  IAGVSPRTVFTPGRRPQSTQSSL----------------VTKSSWTHPVY 63
Arabidopsis thaliana                ANQKKTENESTGGDAAGGNNKGDKGIASYWGVEPNKITKEDGSEWKWNCF 127
Nicotiana tabacum                   KQHDKKVENGGAAASGGGDGGDEKSVVSYWGVPPSKVTKEDGTEWKWNCF 71
Zea mays                            SGSTAAAKAEAVEAAKEGDGKRDKVVSSYWGVAPSKLMNKDGAEWRWSCF 121
Acanthamoeba castellanii            MTDGEPKQTQEEKKAAASNPSIAAQQTVERAQQQSGKSTRVAYTLPHPIW 147
Gregarina niphandrodes              EQGQPKHFNAKSNATASPSLLAESEEYEKN------WVSETRYTQPHPIW 106
Tetrahymena thermophila             ISQSQKQKEVKTQFQPNAVSTEE---------------KLGVYVLPHPIW 80
Paramecium tetraurelia              HLSKLLKKSFSLSTKPNQN-----------------------YTMPHPIW 30
Trypanosoma brucei                  FLETVPVVPLRVSDESSED---------------------------RPTW 65


Ciona intestinalis                  HKQELESVRIS-----HRPPVGKVDKLAYYSVQLLRTGFDVFSGYT---- 179
Strongylocentrotus purpuratus       SEEELDAVEVT-----HNPPKERVDKAAYFACKALRANFDFFSGFS---- 103
Crassostrea gigas                   SEEELHSVKVT-----HKPPEGFVDKLAFRSVKLLRSTFDLLTGFN---- 146
Urechis unicinctus                  SEEELHSVHVT-----HRNPEGIVDKIAYMGVKFTRGCYDFVSGYS---- 162
Nematostella vectensis              --------------------------------------------------
Trichoplax adhaerens                ---------------------------MYSICR----------------- 6
Amphimedon queenslandica            TESELNEVTIT-----HVKPSLFVDKAAYASVQTLRFFFDVFSGYY---- 118
Penicillium rubens                  TEAQMQSIQIA-----HRQTANWSDWIALGTVRFFRWGMDTATGYK---- 129
Aspergillus flavus                  TTSQLHSIQTA-----HRNAIDWSDRMALGTVRFLRWGMDLVTGYH---- 104
Arabidopsis thaliana                RPWETYKADITIDLKKHHVPTTFLDRIAYWTVKSLRWPTDLFFQRR---- 173
Nicotiana tabacum                   RPWETYKADLSIDLTKHHAPTTFLDKFAYWTVKALRYPTDIFFQRR---- 117
Zea mays                            RPWEAYKPDTTIDLNRHHEPKVLLDKIAYWTVKLLRVPTDIFFQRR---- 167
Acanthamoeba castellanii            QNEYVDAVEIN-----HTPPENLTDKLALNTVRLMRFNFDWMSGYS---- 188
Gregarina niphandrodes              NDEEVHAVQKT-----HFRPRGVSDRAALYLLRSIRGVFDVCTGYA---- 147
Tetrahymena thermophila             TKEDVENVQIT-----HFKPKNIGDRLSHYLIQSMRLGFDVMSGYKKVFP 125
Paramecium tetraurelia              NKPELEKVSLE-----HKTAITFGDHFAYYFIQSMRLGFDVMSGYK---- 71
Trypanosoma brucei                  SLPDIENVAIT-----HKKPNGLVDTLAYRSVRTCRWLFDTFSLYR---- 106
```

```
Ciona intestinalis                  LGT------YTGRLDEKQWVKRIIFLETIAGVPGMVGAMVRHLVSLRRLK 223
Strongylocentrotus purpuratus       WGK----------RTERKWIYRIIFLETVAGVPGMVAAMSRHLRSLRRMQ 143
Crassostrea gigas                   WGE----------RTEKKWVLRICFLETVAGVPGMVAAMTRHLHSLRRLK 186
Urechis unicinctus                  RGR----------QDEKMWVSRLCFLETVAGVPGMVAAMVRHLTSLRKMR 202
Nematostella vectensis              -----------------------MLETVAGVPGMIGAMTRHFNSLRRLT 26
Trichoplax adhaerens                ---------------------RIIFLETVAGVPGMVAAMTRHLHSLRRMR 35
Amphimedon queenslandica            IGK------FRGTLNEKKWLTRIIFLETVAGVPGMIAAMLRHLRSLRYLQ 162
Penicillium rubens                  HPKPGEQLPARFKMTEHKWLNRFVFLESIAGVPGMVGGMLRHLRSLRKMK 179
Aspergillus flavus                  HSHPRDAHSPRFRMTEEKWITRFIFLESVAGVPGMVAAMLRHLKSLRRMR 154
Arabidopsis thaliana                YGC------------------RAMMLETVAAVPGMVGGMLLHCKSLRRFE 205
Nicotiana tabacum                   YGC------------------RAMMLETVAAVPGMVGGMLLHCKSLRRFE 149
Zea mays                            YGC------------------RAMMLETVAAVPGMVGGMLLHLRSLRRFE 199
Acanthamoeba castellanii            WGK----------LTEADWLRRIIFLETVAGVPGSVAAILRHLHSLRRLK 228
Gregarina niphandrodes              FGP----------LSAQGWINRVVLLETIAGVPGLVGAAFRHLRSLRRME 187
Tetrahymena thermophila             WQQ------KSGELTERGWLNRMVFLETVAGVPGFVAAMHRHLRSLRRME 169
Paramecium tetraurelia              KTL----PFQSELVSEKKWINRVLFLETVAGVPGFVAGMHRHLRSLRGMK 117
Trypanosoma brucei                  FGS----------ITESKVISRCLFLETVAGVPGMVGGMLRHLSSLRYMT 146
                                                             **  * ***       *  ***

Ciona intestinalis                  RDHGWIHTLLEEAENERMHLMTAMRIANPGIIMRTSIVVAQGIFVSGFSL 273
Strongylocentrotus purpuratus       RDHGWIHTLLEEAENERMHLMTALEIKQPSLFFRLMVLGAQGIFVNMFFI 193
Crassostrea gigas                   RDHGWIHTLLEEAENERMHLMTALQLRQPSWLFRSGVIVSQGAFVTMFSI 236
Urechis unicinctus                  RDHGWIHTLLEEAENERMHLMVMLQLKQPSLFFRLGVMVTQGVFVSGFSV 252
Nematostella vectensis              RDHGWIHTLLEEAENERMHLMTALELKRPGILFRGVILAAQGVFVNMFFI 76
Trichoplax adhaerens                RDYGWIHTLLEEAENERMHLLTALHLKRPGPFFRACVILGQGIFVNFFIL 85
Amphimedon queenslandica            RDHGWIHTLLEEAENERMHLLTALVLRKPGFLFRFAVIGAQGIFVTLFSA 212
Penicillium rubens                  RDNGWIETLLEEAFNERMHLLTFLKLAEPGWFMRVMVIGAQGVFFNGFFL 229
Aspergillus flavus                  RDYGWIETLLEEAYNERMHLLTFLKLSQPGPAMYFMVLAAQCVFFTGFSL 204
Arabidopsis thaliana                QSGGWIKALLEEAENERMHLMTFMEVAKPKWYERALVITVQGVFFNAYFL 255
Nicotiana tabacum                   QSGGWIKALLEEAENERMHLMTFMEVAKPNWYERALVFAVQGVFINAYFV 199
Zea mays                            HSGGWIRALLEEAENERMHLMTFMEVAKPKWYERALVLAVQGVFFNAYFL 249
Acanthamoeba castellanii            RDHGWIHTLLEEAENERMHLLTGLKLKQPGKIFRTAVVVTQGIFFNFFFA 278
Gregarina niphandrodes              RDYGWIHTLLEEAENERMHLMSALMIKNPGRVFRTFVIAGQLFFLPLYTG 237
Tetrahymena thermophila             RDYGWIHVLLEEAENERMHLLTFLKVQKPTLLFRLGVISAQFNYVLMFGL 219
Paramecium tetraurelia              RDQGWIHTLLEEAENERIHLLTFLNIKKPSLIFRTGVVLAQAWYVALFGV 167
Trypanosoma brucei                  RDKGWINTLLVEAENERMHLMTFIELRQPGLPLRVSIIITQAIMYLFLLV 196
                                       ***  ** ** *** **        *           *

Ciona intestinalis                  AYLISPRFCHRFVGYLEEEAVKTYTHCLEELDSGN--LKMWCRMKAPEIA 321
Strongylocentrotus purpuratus       SYLVSPRFCHRFVGYLEEEAVITYTKLLKDLRADA--LPKWKDRIAPEIS 241
Crassostrea gigas                   AYMLSPRFCHRFVGYLEEEAVFTYSKCLKDIESGS--LKHWQTKAAPDVA 284
Urechis unicinctus                  AYMLSPRLCHRFVGYLEEEAVITYTKLLKEIDSGA--MQHWNTLPGPDVA 300
Nematostella vectensis              AYLTSPRFCHRFVGYLEEEAVKTYTYCLECIDNGK--LPTWNTLKAPKIA 124
Trichoplax adhaerens                SYLISPRFCHRFVGYLEEEAVITYTKCLNQIDRGY--LPMWAKMDAPDIA 133
Amphimedon queenslandica            AYIISPKFCHRFVGYLEEEAVKTYTHCLECIDRGD--LKVWAKTAAPSIS 260
Penicillium rubens                  SYLISPRICHRFVGYLEEEAVITYTRAIEELEAGN--LPEWKDLDAPEIA 277
Aspergillus flavus                  AYLISPRICHRFVGYLEEEAVITYTKAIQELDKGN--LPLWSNMEAPAMA 252
Arabidopsis thaliana                GYLISPKFAHRMVGYLEEEAIHSYTEFLKELDKGN--I---ENVPAPAIA 300
Nicotiana tabacum                   TYLLSPKLAHRIVGYLEEEAIHSYTEFLKELDKGN--I---ENVPAPAIA 244
Zea mays                            GYLISPKFAHRVVGYLEEEAIHSYTEYLKDLEAGK--I---ENVPAPAIA 294
Acanthamoeba castellanii            AYLVSPRFCHRFVGYLEEEAVRTYTHLLHDLDAGK--LPEWKDTPAPEIA 326
Gregarina niphandrodes              MYLVSPRLAHRFVGYLEEEAVKTYTHLLEELEAGH--QPELASMKAPLLA 285
Tetrahymena thermophila             LYQFFPRVCHRIVGYLEEEAVKTYTHCIEVINQENSSISHWKTKKAPQIA 269
Paramecium tetraurelia              AYIFWPRVCHRIVGYLEEEAVKTYTHMIHEIEREGSPIHSWTTRKANQNS 217
Trypanosoma brucei                  AYVISPRFVHRFVGYLEEEAVITYTGVMRAIDEGR--LRP-TKNDVPEVA 243
                                     *   *   ** ********   *

Ciona intestinalis                  VEYWKLPDDA-MMRDVILAIRADEAHHRSVNHDLGSR---KP-DEQNPYP 366
Strongylocentrotus purpuratus       INYWKLRPDA-DYIDLFRAIRADEAHHREVNHTLSDI---KP-DDRNPFG 286
Crassostrea gigas                   IRYWKLPETA-SMKDVVLAIRADEAHHRVVNHTLASM---KE-DEYNPYE 329
Urechis unicinctus                  ISYWKLRPGA-AMKDVILAIRADEAHHRVVNHTLSSL---KD-DDYNPYK 345
Nematostella vectensis              SNYWKLKEDA-VMRDVILAIRADEAHHRVVNHTLSSI---HL-DDPNPFF 169
Trichoplax adhaerens                RTYWQLKPDA-KMRDVILAIRADEAHHRLVNHTLASI---NP-EQKNPYK 178
Amphimedon queenslandica            QKYWQLPEGA-MMRDVILAIRADEAHHCEVNHTLSSM---DM-DKQNPFE 305
Penicillium rubens                  VKYWQMPEGQRKMKDLLLFIRADEAKHREVNHTLANL---KPTQDPNPYQ 324
Aspergillus flavus                  IKYWQMPEGQRSIRSLLLCVRADEANHRDVNHTLGNL---NQDSDPNPFS 299
Arabidopsis thaliana                IDYWRLPADA-TLRDVVMVVRADEAHHRDVNHFASDIHYQGRELKEAPAP 349
Nicotiana tabacum                   IDYWRLPKDS-TLRDVVLVVRADEAHHRDVNHFAPDIHYQGQQLKDSPAP 293
Zea mays                            IDYWQLPADA-TLKDVVVVVRSDEAHHRDVNHFASDIHFQGMQLKETPAP 343
Acanthamoeba castellanii            RQYWKMGDDA-KWRDVVALIRADEAHHREVNHTFANL---QL-EQDNPFP 371
Gregarina niphandrodes              RQYWSLKNDA-SFTDMIFAIRADESHHRDVNHTFANM---KP-NEENPFE 330
Tetrahymena thermophila             IDYWRLPENA-TMEDVIYAIRKDEEHHRDVNHDLASD---YSQTKVLADT 315
Paramecium tetraurelia              IEYWGLDENA-TLLDVVKAIRKDEEHHKDVNHYFADD---YTQSKPNPFP 263
Trypanosoma brucei                  RVYWNLSKNA-TFRDLINVIRADEAEHRVVNHTFADMHEKRLQNSVNPFV 292
                                      **                *  **  *  ***
```

```
Ciona intestinalis                 ----------PGQ------------------------- 369
Strongylocentrotus purpuratus      ----------PGE------------------------- 289
Crassostrea gigas                  ----------PGK------------------------- 332
Urechis unicinctus                 ----------PGQ------------------------- 348
Nematostella vectensis             ----------PGQRKL---------------------- 175
Trichoplax adhaerens               ----------PGE------------------------- 181
Amphimedon queenslandica           ----------PGK------------------------- 308
Penicillium rubens                 IEYADLSVSHPTKGIDNLRPEGWDRNEIFMGKARTEKS 362
Aspergillus flavus                 AKFRNALKE-ASQPLSPVKEHR---------------- 320
Arabidopsis thaliana               ----------IGYH------------------------ 353
Nicotiana tabacum                  ----------IGYH------------------------ 297
Zea mays                           ----------IEYH------------------------ 347
Acanthamoeba castellanii           ----------PGH------------------------- 374
Gregarina niphandrodes             ----------PGH------------------------- 333
Tetrahymena thermophila            ----------TQDEHYI--------------------- 322
Paramecium tetraurelia             ----------PGK------------------------- 266
Trypanosoma brucei                 VLKKNPEEMYSNQPSGKTRTDFGSEGAKTASNVNKHV- 329
```

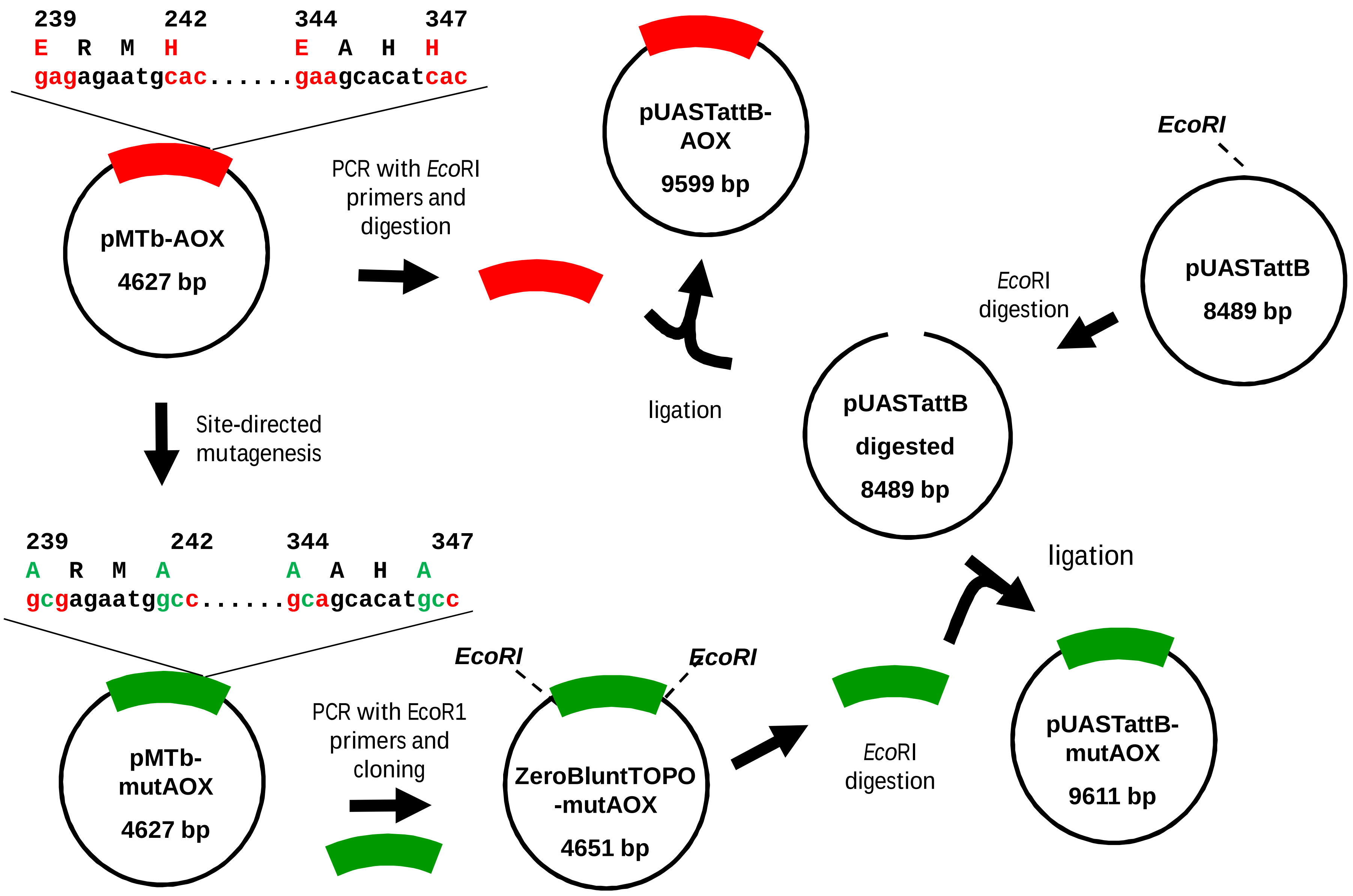

Andjelković et al., Figure S2

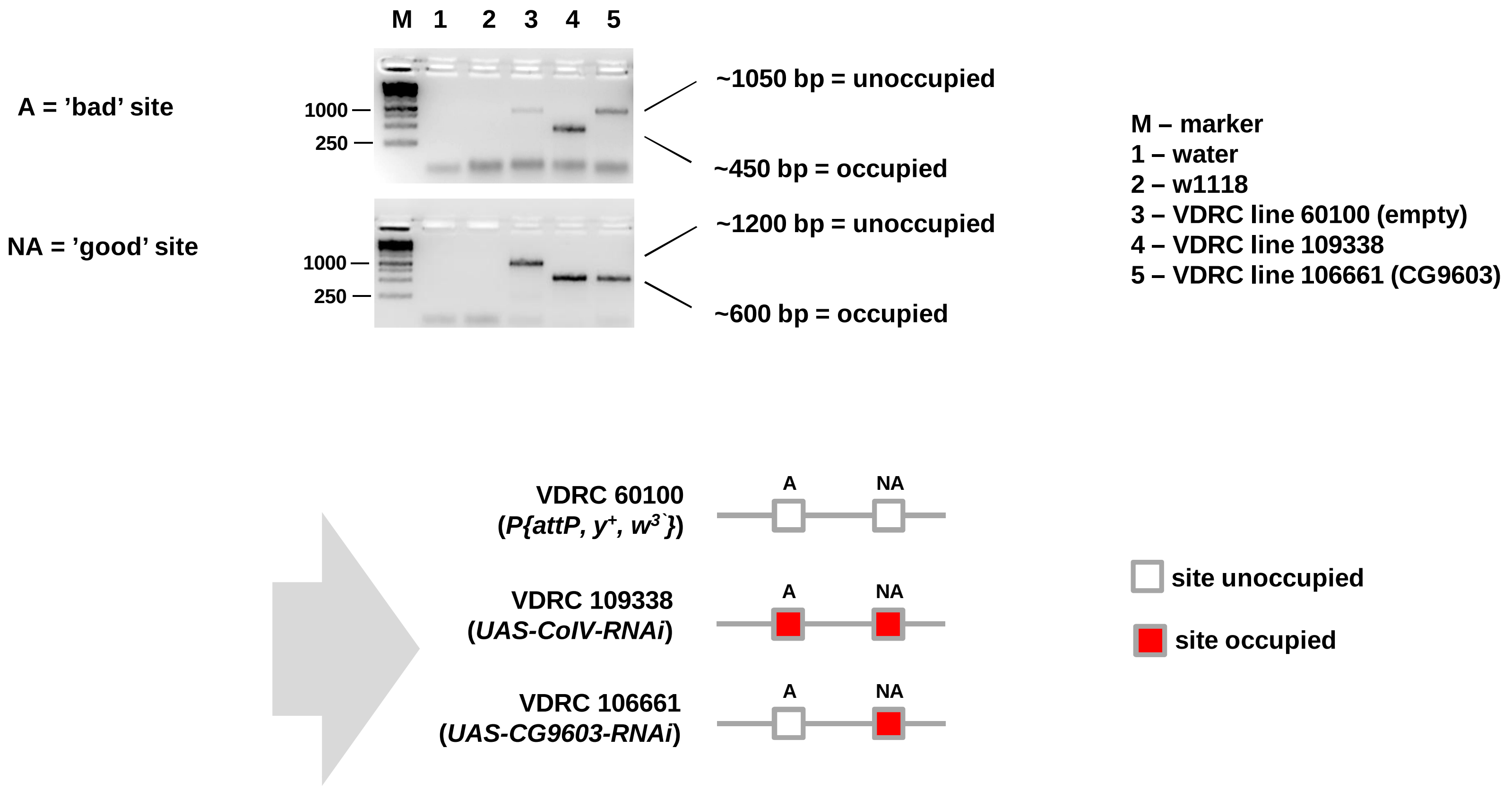

Andjelković et al., Figure S3
